# A Comprehensive Review of Micro UAV Charging Techniques

**DOI:** 10.3390/mi13060977

**Published:** 2022-06-20

**Authors:** Syed Agha Hassnain Mohsan, Nawaf Qasem Hamood Othman, Muhammad Asghar Khan, Hussain Amjad, Justyna Żywiołek

**Affiliations:** 1 Optical Communication Laboratory, Ocean College, Zhejiang University, Zheda Road 1, Zhoushan 316021, China; 2 School of Telecommunications Engineering, Xidian University, Xi’an 710071, China; nawaf102005@gmail.com; 3 Electrical Engineering Department, Hamdard University Islamabad, Islamabad 44000, Pakistan; m.asghar@hamdard.edu.pk; 4 Satellite Communication and Networking Laboratory, Ocean College, Zhejiang University, Zheda Road 1, Zhoushan 316021, China; 21934205@zju.edu.cn; 5 Faculty of Management, Czestochowa University of Technology, 42-201 Czestochowa, Poland; justyna.zywiolek@wz.pcz.pl

**Keywords:** wireless power transfer, unmanned aerial vehicles (UAVs), charging, battery capacity, laser power transfer (LPT)

## Abstract

The groundbreaking Unmanned Aerial Vehicles (UAVs) technology has gained significant attention from both academia and industrial experts due to several applications, such as military missions, power lines inspection, precision agriculture, remote sensing, delivery services, traffic monitoring and many more. UAVs are expected to become a mainstream delivery element by 2040 to address the ever-increasing demand for delivery services. Similarly, UAV-assisted monitoring approaches will automate the inspection process, lowering mission costs, increasing access to remote locations and saving time and energy. Despite the fact that unmanned aerial vehicles (UAVs) are gaining popularity in both military and civilian applications, they have a number of limitations and critical problems that must be addressed in order for missions to be effective. One of the most difficult and time-consuming tasks is charging UAVs. UAVs’ mission length and travel distance are constrained by their low battery endurance. There is a need to study multi-UAV charging systems to overcome battery capacity limitations, allowing UAVs to be used for a variety of services while saving time and human resources. Wired and Wireless Power Transfer (WPT) systems have emerged as viable options to successfully solve this difficulty. In the past, several research surveys have focused on crucial aspects of wireless UAV charging. In this review, we have also examined the most emerging charging techniques for UAVs such as laser power transfer (LPT), distributed laser charging (DLC), simultaneous wireless information and power transfer (SWIPT) and simultaneous light wave information and power transfer (SLIPT). The classification and types of UAVs, as well as various battery charging methods, are all discussed in this paper. We’ve also addressed a number of difficulties and solutions for safe operation. In the final section, we have briefly discussed future research directions.

## 1. Introduction

**Figure 1:**
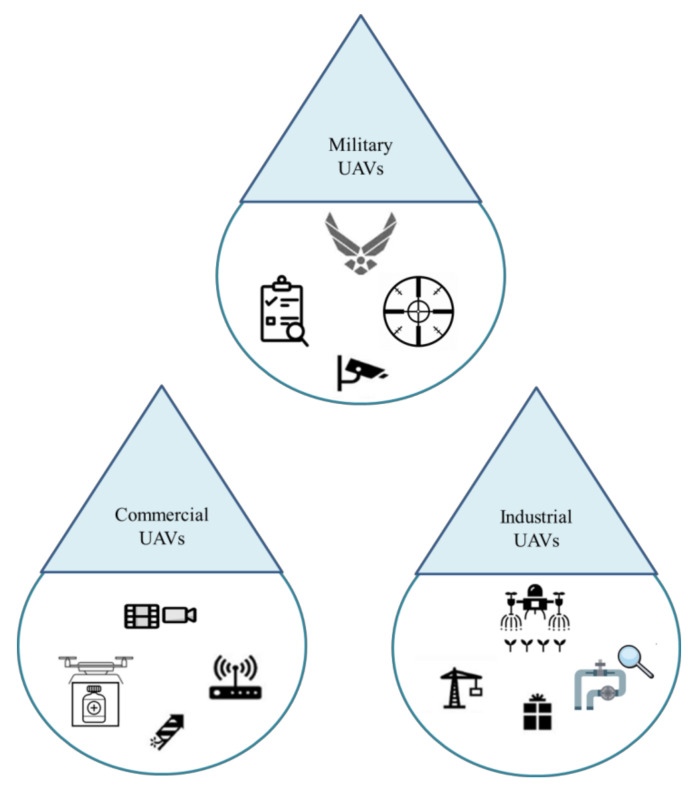
Applications of drones in diverse sectors.

In the last decade, there has been a rapidly growing advancement in the applicability of drones (also known as unmanned aerial vehicles (UAVs)) in different areas, including monitoring, public security, traffic surveillance, military operations, exploration of hidden or hazardous area, damage assessment, 3D mapping, indoor/outdoor navigation, disaster relief, precision agriculture, data sharing, infrastructure management and logistics, [1] owing to their lightweight, compact design and high maneuverability. Figure 1 shows different applications of drones. UAVs carrying a range of technologies for communication and sensing have captured the interest of several service providers including Walmart, DHL, Google and Amazon [2,3]. Due to the extensive increase in online shopping, users now require high-speed delivery services. However, fast delivery is becoming a successful tool for online retailers. Aside from that, retailers may face a critical challenge in providing eco-friendly, cost-effective and efficient last-mile delivery. In this regard, UAVs have emerged as promising solutions with fast and innovative concepts to ensure last-mile delivery along with environmental security. Moreover, drones have become a key element in 5G networks, either as a base station or a mobile relaying system to enable cellular UAV networks. With such an expanding and staggering market interest, service reliability of integrating UAVs will become a critical success factor. However, there are several crucial factors which limit the performance of UAVs. Some of these factors are limited battery endurance, restricted mobility, limited autonomy and limited flight time. Limited flight time is due to different elements, including sensor accuracy, harsh atmospheric conditions, fixed-wing size and battery endurance. Several studies have focused on multiple aspects of UAVs [4] and suggested viable solutions to overcome these challenges, such as using high-quality devices including batteries, wing, geometry, manufacturing materials and motors. Some studies have reported optimization algorithms to seek the shortest route for UAVs to reach their intended destination. 

**Figure 2:**
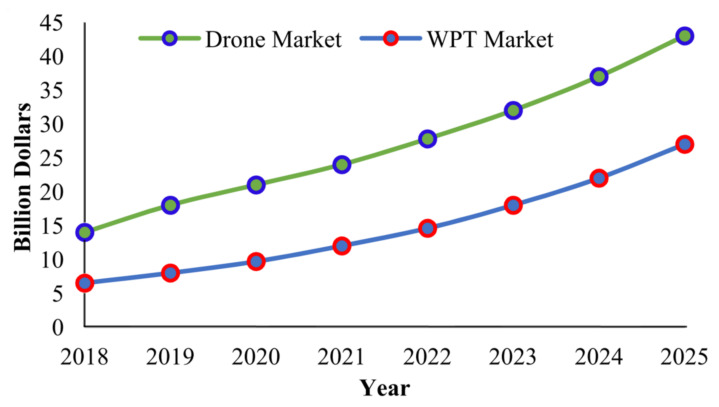
Statistics of Drones and WPT market growth [7].

Figure 2 shows that the UAV market is expected to reach $43 billion in total sales by 2025. UAVs are intended to be controlled remotely, either through predefined trajectories or through a radio controller. UAVs are now essential components in both civilian and military applications. UAVs, on the other hand, are energy-hungry devices that deplete batteries within minutes of operation. Due to limited battery capacity, UAVs have short flight times. In most cases, the traditional battery swapping method is used, in which a drone makes frequent trips to a charging station where the depleted battery is removed and a fully charged battery is installed. However, this physical battery swapping method necessitates human assistance, affecting UAV operations and causing significant service disruptions in remote areas.

These charging techniques are divided into two categories on the basis of transmission distance range: near-field transmission and far-field transmission. Table 1 summarizes different charging techniques to charge UAVs. The energy limitation of UAVs is also addressed through motion control functions and optimization algorithms to enhance energy efficiency [5,6]. However, these efforts cannot fully eliminate these issues as drones are still required to visit charging stations when their batteries are drained. Furthermore, solar-powered drone technology has been proposed to harvest energy from the sun. However, this solar power feature cannot be embedded in small drones. This solar energy harvesting feature also depends on flight weather conditions e.g., drones cannot properly harvest energy at night or on cloudy days, which leads to the UAV’s substandard performance. 

## 2. Related Works

**Figure 3:**
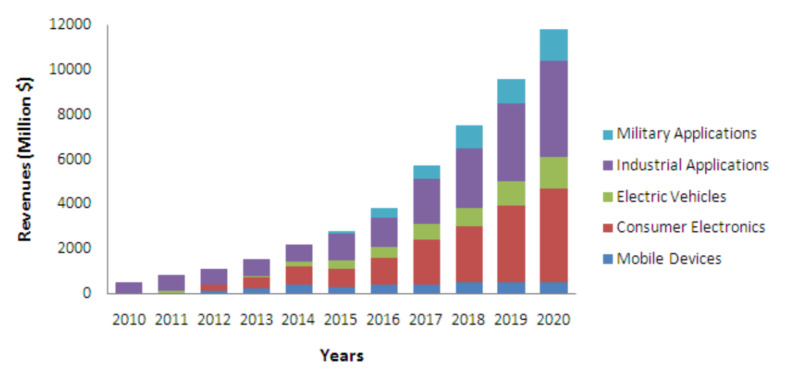
Predicted values of WPT technology in 2020 [16].

Despite all the appealing applications of UAVs, battery-powered UAVs have a major drawback in limited flight duration. This challenge occurs due to the limited capacity of lithium-ion polymer (LiPo) batteries. Most of the previous reported works on UAV issues, such as resource allocation and 3D trajectory optimization, focuses on energy consumption when communicating, data processing, hovering or flying. Several research studies have focused on designing energy-efficient operational mechanisms such as UAV location or path optimization, battery capacity increase and battery hot-swapping to address the energy limitation issues. These mechanisms include communication and trajectory planning for UAVs [6,8]. UAVs consume their battery power during communication, hovering and flying. However, UAVs consume more energy during hovering and flying compared to communication tasks. Thus, the research fraternity has focused on investigating optimization strategies to reduce flight trajectories [9,10]. One approach that is being used to recharge is tethered UAVs [11]. In this technique, UAVs are linked to a base station through a cable to receive power supply continuously. However, this method is only useful in those locations where base stations are located and it also restricts the mobility of the UAVs. Another widely used approach to recharge UAVs is battery swapping [12]. In this technique, UAVs are required to fly back to the ground base stations to replace drained batteries with fully charged batteries. This approach is better than tethered UAVs because it is faster. However, this approach raises some serious concerns, such as the need for human assistance to replace batteries and the disruption of the UAV mission due to the need to leave the operational location to swap batteries. Another technique is called battery dumping, in which UAVs are equipped with many batteries and an individual battery is dumped when it is drained. This technique enhances the flight time by reducing the payload of UAVs. Another method of recharging UAVs is known as gust soaring where UAVs gain energy from airflow and wind through the principles of dynamic soaring. To overcome mobility and deployment location challenges, WPT techniques have emerged as promising alternatives. Researchers and industrial experts have focused on different trials, activities and projects related to WPT techniques. For instance, a New Zealand-based startup company, Emrod, has designed a high power, long-range WPT system to deliver wireless electricity to consumers without requiring copper power lines [13]. Following this idea, the Powerco company started trials of this technology in 2021 [14]. Furthermore, Laser-Motive, a US based company, demonstrated laser-based WPT to fly a drone for more than 12 h [15]. According to [16], it is envisaged that WPT will generate 12 billion dollars of revenue in 2020. This prediction can be seen in Figure 3. In the view of a 2020 Bloomberg report, the expected market value of the global drone wireless charging and infrastructure market for drones will hit USD 249.3 million by 2024 [17]. This is due to diverse applications in the electronic industry with several key benefits in terms of safety, convenience, reliability and a fully automated charging mechanism.

The expanding demand for UAVs WPT technologies is clearly validated by their several applications, such as parcel delivery, medical assistance, traffic monitoring, power line inspection, remote sensing, forest monitoring, law enforcement, search and rescue, precision agriculture and disaster management. Far-field WPT techniques have proven stature to support these applications due to mobility, long distance, non-LoS operations and location flexibility. Generally, these technologies are useful for low-powered devices due to their limited energy efficiency. Despite these issues, several studies have reported far-field WPT techniques for UAVs [18]. In this regard, WPT systems to recharge UAVs through optical energy transfer [19] and RF WPT [20] have been proposed in the literature. In [20], the authors propose a relaying system through time switching and power splitting architecture to harvest both energy and information for UAVs. In the proposed system, the authors optimized UAV system parameters and deployment location for throughput maximization and prolonging of lifetime. While in [19], the authors used an optical beam for UAV communication and charging simultaneously. The authors claimed a high network throughput and a 25% increase in hovering time. However, the proposed system faces limited distance as UAVs must remain in close proximity to the terrestrial base station. To overcome this issue, the authors in [21] suggested terrestrial UAVs (tUAVs) with omnidirectional antennas. However, omnidirectional antennas waste energy in all directions rather than only in the intended direction of the energy receiver. Powering through laser beaming supports longer UAV flight times [22,23]. Laser beaming can be implemented through a laser array oriented using mirrors towards an intended UAV. Moreover, a practical and cost-efficient system for UAVs has been proposed as distributed laser charging (DLC) [24] using PV cells rather than lenses so that the receiver UAVs do not solely depend on laser charging due to LoS path limitations. To overcome this issue, a hybrid approach can be used for lighter UAVs containing small batteries. 

## 3. Battery Charging Techniques for UAVs

Drones are currently used in a variety of applications, including military, power line monitoring, forest monitoring, disaster prevention and smart agriculture [25,26]. Drones carry different types of payloads such as GPS, infrared cameras, batteries and sensors as delivery vehicles. These drones usually carry high energy batteries, e.g., lithium batteries, which support flight times of 20–40 min [27]. However, range and endurance are critical challenges in UAVs due to the limited battery capacity. It is not feasible to increase the battery size of UAVs because it will increase weight, which is another critical concern. Several research studies have addressed battery charging of UAVs, but it requires an intensive investigation by the research fraternity. Jawad et al. [25] suggested three ways to enhance flying time: (i) Drones can be equipped with high battery capacity but it can increase the drone weight. (ii) Battery swapping can be achieved after landing the drone. However, this also causes complexity and high cost when swapping the system. (iii) Recharging can be done at the base station of the drone. Charging can be achieved through a wired or wireless power transfer (WPT) system [28]. 

### 3.1. Battery-Powered UAVs 

Small drones are typically battery-powered and batteries are critical components of these drones [29]. They improve the flexibility and simplicity of the propulsion system. Moreover, battery-powered drones offer cost-effectiveness and enhanced flight time. However, small UAVs have limited battery endurance due to low weight carrying capacity and can travel up to 90 min on LiPo batteries [30]. Small scale UAVs are mostly suitable for commercial applications. Furthermore, Lithium batteries are installed in small UAVs due to their high energy and light weight. Table 2 illustrates a comparison of different batteries. Donateo et al. [31] evaluated different batteries on the basis of state of charge (SOC) for a desired operation. Similarly, the author in [32] investigated parameters which impact the performance of battery-powered UAVs. Battery-powered UAVs face the critical challenge of reduced autonomy. Several research studies have focused on enhancing battery performance to extend UAV mission time. Moreover, safety and stability concerns have been reported as a result of energy density enhancement [33]. Thus, several approaches have been proposed to overcome these limitations, such as super capacitors, solar cells, fuel cells and hybrid charging technologies. 

### 3.2. Battery Swapping

**Figure 4:**
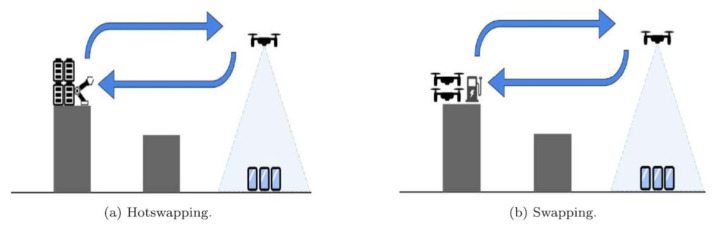
Swapping vs. hot swapping technique [42].

To overcome the limitation of battery capacity, some studies have proposed a battery swapping method to recharge drained batteries of UAVs [35]. The battery swapping method is partially autonomous. It is conducted with human assistance or automatically. In the literature, two techniques have been reported for battery swapping: swapping and hot swapping [36]. In hot swapping, drained batteries are replaced with fully charged batteries while the UAV keeps operating. Hot swapping offers the benefit of allowing the UAV to activate and rejoin its operation. While in swapping, UAVs with drained batteries are replaced with another UAV with fully charged batteries. Figure 4 presents an illustration of swapping and hot swapping techniques. Battery swapping techniques consist of three components: (i) a battery swap station; (ii) UAV swarms; and (iii) a control system for managing UAV swarms. Battery swap stations include contact mechanism, landing infrastructure, onboard circuits and ground electronics. In [37], the authors presented an economic evaluation for recharging of UAVs by considering system complexity, coverage and cost. Multi-UAV systems and cooperative algorithms are proposed to offer continuous service by UAVs. Similarly, the authors in [38] proposed an automated system to quickly swap a depleted battery of a UAV along with charging several other batteries. In [39], the authors reported an automated method to swap UAV batteries to support persistent UAV missions. The proposed dual-drum structure enables the efficient swapping of eight batteries. In [38], the authors briefly discussed the design mechanisms of autonomous swapping stations. In [40], the authors demonstrated a swapping system for accurate landing, UAV healthy monitoring and energy management. In a recent study [41], the authors proposed a patrolling algorithm to optimize UAV trajectory and battery swapping. Furthermore, battery swapping techniques also cause some serious concerns, such as GCS feasibility, landing issues, charging/discharging time, limited applications, increased swapping agents, high cost and system complexity.

### 3.3. Dynamic Soaring

**Figure 5:**
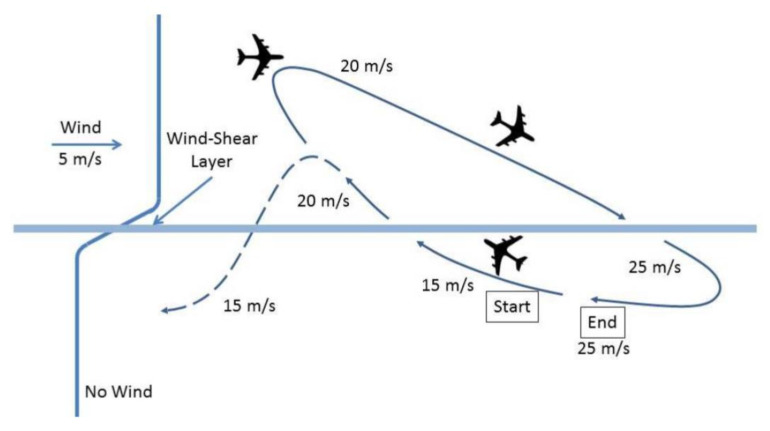
Dynamic soaring maneuver [44].

Dynamic or gust soaring is based on the behaviour of albatrosses and prolongs UAV mission duration [43]. The key principle is to gain energy from airflow and wind. Figure 5 presents an illustration of dynamic soaring maneuvering of an albatross. Figure 5 shows that an albatross can gain wind velocity without wasting energy through the alignment of its body and wing position. Dynamic soaring is useful to attain velocity from ambient energy sources without discharging the battery of a UAV. Consequently, it will enhance the durability of UAVs. However, there are several critical concerns related to dynamic soaring. The major drawback is that dynamic soaring is only suitable for fixed-wing UAVs that have sufficient wing geometry to harness the energy from the wind. However, this technology is not applicable to multi-rotor drones due to varying geometries. Another major drawback is the intermittent nature of wind. Thus, the drone will not be able to use this feature in the absence of wind as it is highly dependent on these environmental conditions. Furthermore, to effectively mimic the maneuver and harness maximum energy, fixed-wing UAVs must be equipped with a sophisticated control system.

### 3.4. Tethered UAVs 

**Figure 6:**
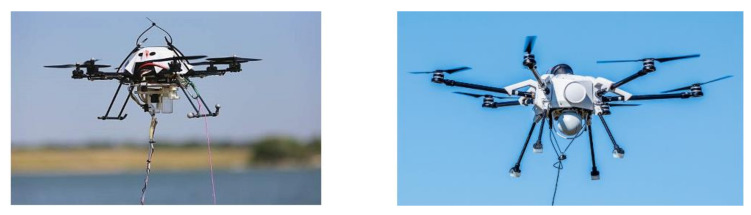
Tethered UAVs [50,51].

Tethered UAVs can support unlimited autonomy. This technology does not require physical landing on a charging station and avoids repeated recharging. In this technology, UAVs can get a continuous power supply through a connecting cable from a charging station. This technology ensures efficient and safe data transmission. In general, copper wires are used as power supply lines. However, optical fiber technology is also used in the tethered UAV area. Fiber optic cable supports kilowatts of power transfer using high intensity optical beams. Optical fiber technology can significantly reduce the UAV payload and power lines compared to copper lines [45]. Two examples of tethered UAVs are presented in Figure 6. Muttin [46] proposed a maritime application-based tethered UAV for detecting oil pollution from ships. Moreover, Gu et al. [47] demonstrated a tethered UAV for nuclear power plants to support extremely long-endurance operations. In [48], the authors proposed a method for UAV localization in an indoor environment using a quasi-taut tether. In a recent study [49], the authors used UAV tethered technology for 3D deployment of aerial base stations. 

### 3.5. Charging from a Power Line 

Power lines are abundant in both rural and urban areas. These power lines can be used as a source of power and electricity supply. Several research studies have investigated tethering UAV technology to power lines to deliver the required energy to UAVs. The technique is viable to efficiently transmit data and power to UAVs. In [44], authors proposed the recharging of UAVs through power lines. An important technology to implement this technology is known as power line perching. It is based on landing a UAV on a current carrying conductor to recharge itself. It involves two aspects. Firstly, the process of guiding the UAV to the location of the power lines. Secondly, the charging process to deliver power to the UAVs. In addition, the conversion of high voltage energy from power lines into low voltage for the UAV’s battery must be taken into account. In the context of landing maneuvers, it is worth noting that navigational electronics reading will be highly affected by the EM field. Researchers from the Massachusetts Institute of Technology (MIT) proposed a solution to overcome this challenge [52,53]. The authors developed an internal measurement unit within the UAV which can guide it according to the EM field of power lines. The proposed mechanism involves a particle filter and synchronous demodulation. Though this proposed mechanism is promising, it involves high computational power and complex signal processing. 

### 3.6. Fuel Cell (FC)

**Figure 7:**
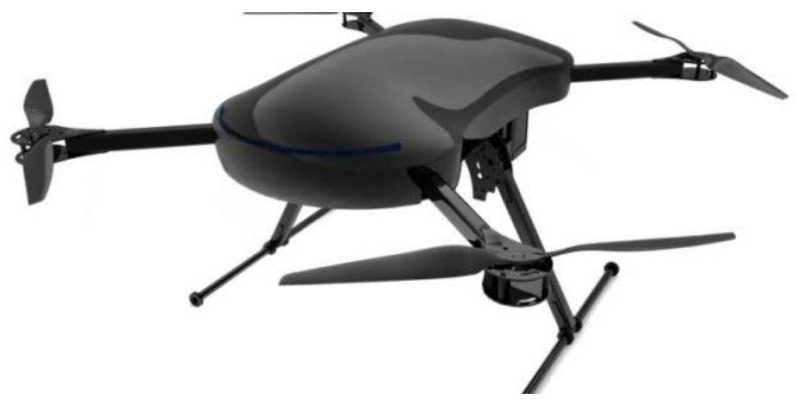
Hydrogen-powered UAV [60].

To achieve long missions and enhance the battery endurance of UAVs, researchers have suggested the implementation of fuel cells (FC) technology. A typical UAV LiPo battery has 250 Wh/kg of energy, whereas compressed FC has 1000 Wh/kg of energy [54]. Furthermore, refueling in FC is faster and more efficient than battery charging and swapping technology [55]. Several technologies are being implemented in the FC industry. Most of these are based on temperature characterization, electrolytes, catalyst and chemical criteria. In [56], Sharaft et al. evaluated the performance of frequently used FCs in UAVs, e.g., Solid Oxide Fuel Cell (SOFC), Methanol Fuel Cell (MFC) and Proton Exchange Membrane Fuel Cell (PEMFC). It can be concluded that the most frequently used FCs in UAVs are PEMFC. Several leading research institutes have dedicated their efforts to FC technology. Georgia Tech University experimentally demonstrated a 500 W FC-powered UAV system, while the Office of Naval Research (ONR) set a breakthrough record for an FC-powered UAV for the highest endurance of 48 h [57]. Furthermore, a Korean aerospace research institute demonstrated an FC-powered UAV with landing, vertical takeoff, monitoring and hovering capabilities [58]. Although FC technology has appealing advantages, such as reduced power losses and reduced carbon dioxide, FC technology is hampered by a sluggish dynamic response causing voltage fluctuations [59]. Moreover, absorption of excessive energy is difficult, which leads to a cold start. Similarly, storage of some FC, such as Hydrogen FC as shown in Figure 7, under low temperature and high pressure is another critical concern. 

**Figure 8:**
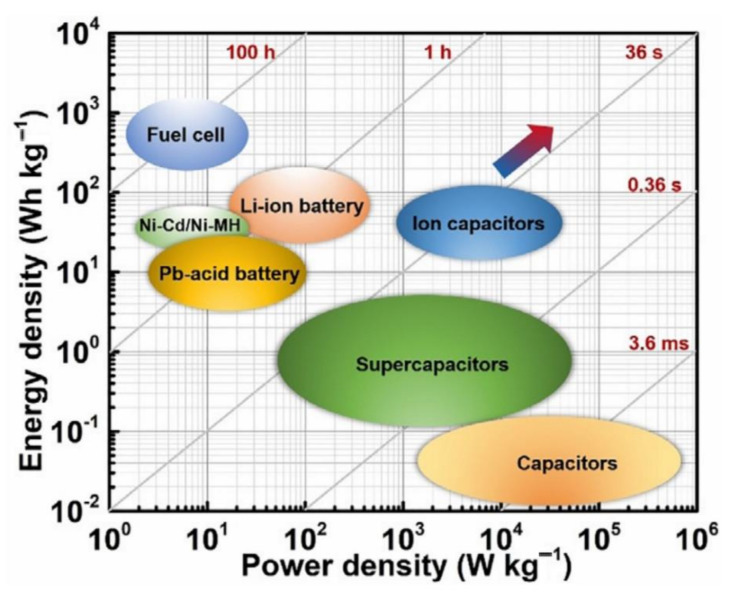
The comparison of energy density and power density for different energy storage devices [63].

In [61], Cai et al. briefly discussed different types of batteries, such as Ni-Mh, Ni-Cd and Li-ion. Authors carried out a comparison of these batteries with fuel cells considering different parameters such as endurance, efficiency, temperature effect, discharging and power densities. Figure 8 presents a comparison of FCs, super capacitors and batteries considering power density vs. energy density aspects. It clearly indicates that FCs outperform other resources in terms of energy density. In [62], the authors investigate a drone for mobile crane inspection. As the flight time of drones is short for commercial applications, the authors used lithium-ion batteries and proton exchange membrane cells to overcome this short flight time. The authors carried out analysis of both the environmental impact and the economic point of view. Lightweight fuel cells resulted in being more expensive than Li-ion batteries. 

### 3.7. Super Capacitor (SC)

Battery-powered UAVs are subject to high energy density, sluggish response and limited lifespan. On the other hand, FC-powered UAVs are subject to sluggish dynamic response, which can cause voltage instabilities. In such cases, super capacitor technology emerges as a promising alternative to UAVs with a faster energy storage capability. This is due to the fact that SCs are characterized by reduced voltage instability, high lifespan, low maintenance cost, charging tolerance, wide operating temperature range, low energy density, fast response and higher energy [64]. The SC offers various benefits in terms of reinforced power density, rapid response of energy management system (EMS) and supplying architecture. Several research studies have focused on integration of SCs into UAVs. In [65,66], the authors carried out critical analysis of a hybrid UAV propulsion system containing FC, FC and battery. These studies indicate a good performance of SC on system load stability and dynamic response. Some studies [67,68] dedicated to hybrid UAV models indicate a significant contribution of SC in absorbing power instability and supplying peak power. Table 3 summarizes a comparison of the main characteristics of SCs and batteries. 

### 3.8. Photovoltaic Cell-Based UAV Charging

PV cells are commonly considered to charge the batteries and enhance the flight time of UAVs. PV cells make use of sunlight to charge UAV batteries. Both the PV cells and the batteries utilized in a UAV are used in this way. Whenever sunlight is present, the required power is provided by PV cells and in the absence of sunlight, batteries are used to deliver the required power to the UAV. Research work on solar-powered UAVs reported that several parameters play key roles such as temperature intensity, angel of incidence of sunlight, geometry and the position of PV cells [70]. This technique is not suitable in scenarios with insufficient sunlight. There is a need to adopt alternative strategies whenever sunlight is not present to continue UAV flight. These methods include additional power supplies, enhanced battery power or PV cell size and automatic position adjustment according to sun position. As PV cells require a certain payload capacity and wing length of the UAV, it is feasible for fixed-wing UAVs. Moreover, environmental conditions such as humidity, temperature, fog and clouds reduce the system efficiency or reliability. 

**Figure 9:**
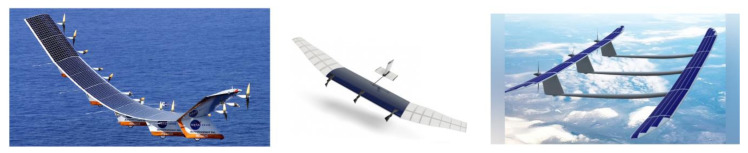
Solar-powered UAVs [75,76,77].

Solar energy-based UAVs have gained the attention of many researchers. Figure 9 presents solar-powered UAVs. Solar-powered aircrafts are currently being used for power line inspection, forest fire fighting, border surveillance and high-altitude communication. This technology can be used to supersede environmental, scientific and communication satellites used for military and civilian applications. Sufficient flight power can be achieved through the appropriate selection of PV cells and taking into account the efficiency and weight is the key to ensuring long flight times and high endurance in solar-powered UAVs. Different solar cells and materials are used to attain cost-effectiveness and high efficiency. Some studies suggest mono-crystalline silicon PV cells due to their affordable cost and high efficiency [71]. Furthermore, mono-crystalline silicon PV cells offer high flexibility which supports easy integration in the UAV wing. It is still important to investigate PV cells with novel designs, energy management strategies and manufacturing materials to ensure high efficiency and high availability [72]. The research fraternity should focus more on material science developments as low photoelectric efficiency is a major limiting factor in this area [73]. Some researchers proposed an effective approach for the optimization of solar-powered UAV flight trajectories to gain more solar radiation, along with low consumption of mechanical energy [74].

## 4. Wireless Power Transfer (WPT)

UAVs have gained significant attention due to their diverse applications in the electronic industry with several key benefits in terms of safety, convenience, reliability and a fully automated charging mechanism. These benefits can be attained through different WPT techniques. Another key feature of WPT is that it is very essential in different environments where wired power transfer techniques are dangerous, difficult, or impossible, such as the underwater environment and high voltage power applications. The research fraternity has been exploring trade-offs and evaluating different WPT techniques. These techniques are categorized as: radiative electromagnetic (EM) and non-EM techniques. In non-EM, power is transferred using acoustics or optical sources such as lasers. EM is further classified into radiative far-field, non-radiative and mid-field radiative and non-radiative. In radiative power transmission, RF is used, while in non-radiative power transmission, capacitive coupling, inductive coupling and magnetic resonance coupling are used. Currently, these WPT techniques are commercially available for different applications such as smart phone charging and charging implantable medical devices, etc. and are still being developed for different applications. The aforementioned WPT techniques ensure efficient and reliable wire power transmission between UAV and base station. The application of WPT technology to UAVs should take into account some critical issues such as misalignment, interference and payload. UAV-based WPT system must be lightweight to avoid payload reduction. Furthermore, the WPT techniques must ensure misalignment tolerance between coils, high landing precision and efficient power transfer. Among these challenges, misalignment is a dominant issue as landing accuracy is low in the case of UAVs. Consequently, it affects coupling factor, transfer efficiency and power transfer. Several research studies have reported these major issues concerning recharging UAV batteries through WPT techniques [78,79]. UAV charging can be achieved through inductive coupling WPT to enhance both range and flight time for inspection, monitoring and surveillance tasks. UAV-empowered inspection techniques can overcome several limitations of existing inspection techniques, such as expensive task and hazardous operation through manned helicopters. In [80], the authors demonstrated magnetic resonance coupling (MRC) WPT to recharge drones and tested the proposed system with different distances and misalignment topologies. The authors reported 90% transfer efficiency at 10 cm distance. Moreover, Junaid et al. [81] presented a vision-based, closed-loop target detection through UAV for outdoor applications. The authors designed a charging station which prolonged the flight time and enhanced the endurance of a UAV. In [82], Blain proposed a novel mid-air inductive charging mechanism to charge multiple drones at the same time without any need for landing using global energy transmission (GET). Most studies on charge scheduling of UAVs consider a centralized architecture. Only a few works have been reported through the peer-to-peer network on UAVs charging through blockchain technology [83,84]. Next, we discuss laser power transfer (LPT) in detail.

### 4.1. Laser Power Transfer (LPT)

**Figure 10:**
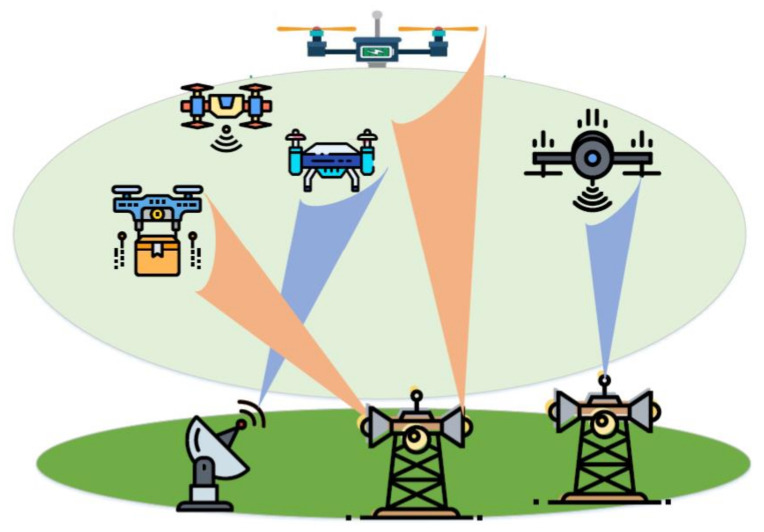
Recharging of multiple UAVs through laser beams [89].

Laser power transfer is another method used to charge UAVs, mostly in space and military applications [85]. With this charging technique, laser beams of specific wavelength and frequency feed PV cells mounted on the UAV. These PV cells are used to harvest energy from the laser transmitter to power the UAV and charge its batteries. Laser beaming technique is used for rotary wing and fixed-ing UAVs. It has become a viable solution to unlimited endurance. It has the capability to deliver high energy to the receiver using narrow beam divergence [23]. Laser power transfer is envisaged to empower several energy-hungry missions of UAVs over long distances. Some studies [86,87] have reported on the feasibility of laser power transfer for UAVs. In [88], the authors presented results pertaining to current, voltage and efficiency based on laser wavelength in a laser PV cell and material type. In [24], the authors discussed results pertaining to wavelength and temperature output of PV cells used to charge the system through laser beams. In [87], the authors proposed a controller design strategy on the basis of laser PV module output characteristics to control the power converter of a laser power transfer system. Some critical concerns are blockage, mobility and performance in long-range flights. This method is restricted in certain areas, such as military areas and airports and other scenarios where laser beams are hazardous to human health and living environments. Figure 10 illustrates an overview of a multi-UAV WPT system using laser beams. 

#### 4.1.1. PV Cell Selection

**Figure 11:**
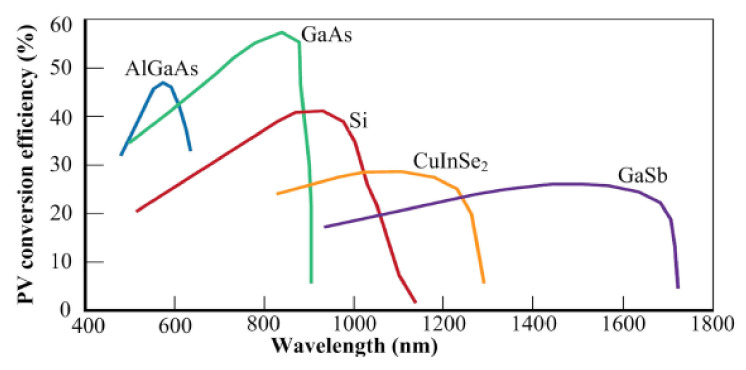
Spectral response of PV materials [90].

The receiver component of any LPT system should be carefully designed for the efficient conversion of optical signals into electricity. The most common conversion technologies are photovoltaic, pyroelectric and thermoelectric. Among these technologies, photovoltaic technology is considered the most mature and delivers the highest efficiency when delivering high power at longer distances. For efficient performance, laser power, wavelength, PV cell material and temperature should all be taken into account. For any PV cell receiver, the photon energy must be equal to or higher than the bandgap energy of the material. This photon energy is proportional to its frequency. Hence, a monochromatic light with an ideal frequency is considered an ideal light source. Figure 11 illustrates the spectral response of various PV cells [90]. 

It can be seen from the graph curves that commonly available GaAs and Si PV cells give the highest conversion efficiencies at 850 nm and 900 nm, respectively. While AIGaAs gives the highest response from 550–600 nm. A PV cell gives efficient response for a given temperature and generates more power if it is illuminated with an intense light beam [91]. Thus, PV cells operate efficiently at high laser power intensity. Generally, GaAs [92] and Si [93] are often used, but it is also important to develop the optical receiving device according to the wavelength of the optical source in order to enhance the overall efficiency of the system. 

#### 4.1.2. Laser Selection

The American National Standard for Safe Use of Lasers (ANSI Z136.1) [94] gives guidance for the installation, services, maintenance and safe use of lasers for optical communication systems. It serves as the basis for laser selection and safety programs throughout the USA, including academic laboratoriesand the government and market sectors. This document includes laser selection, working, standard operating process and safety features to protect users from any possible hazards. Several types of laser are available on the market nowadays. Proper selection of the laser is crucial for OWPT systems. In principle, laser selection should comply with various constraints such as: (1) OWPT through the atmosphere or different environment; and (2) the maximum required transfer energy for the OWPT system. One constraint is related to laser selection with proper wavelength. As the atmosphere contains several gases which fluctuate according to environment conditions, so th atmosphere can absorb laser light with specific wavelength. For the efficient performance of an OWPT system, lasers should have the capability to operate with maximum energy transfer. K. J. Duncan reported laser-based component selection in which the 780 nm and 1100 nm regions are suitable for commercially available laser technologies [95]. The spectral window of 800, 900 and 1500 nm is useful for power beaming to UAVs. Other important factors are laser weight, size, laser safety, component efficiency, working under harsh conditions, laser divergence and maturity of laser technology. The key features of lasers include:

1. *Directionality:* typically, the laser beam gives a low divergence.
2. *Coherence:* The emitted photons are coherent and carry a constant phase relationship.
3. *Monochromaticity:* laser beams contain a narrow range of wavelengths.

## 5. Distributed Laser Charging (DLC) for UAVs

As can be seen by the transformation of electronic devices into wearable devices, desktops into tablets and laptops, and desk phones into cell phones, mobility has become a major issue for these devices. Internet of things (IoT) and mobile devices like smartphones and sensors, and emerging commercial technologies such as UAVs, are powered by batteries with limited operation times. IoT sensors, especially those deployed in harsh environment such as underwater and in volcanoes, are difficult to charge. UAV battery swapping also causes disruptions in continuous missions. Meanwhile, the charging of mobile devices such as smartphones, laptops, tablets and wearables involve the difficulty of finding a power socket and carrying a power cord to recharge any device also incurs disruption. Therefore, the concept of charging mobile devices, anytime, anywhere has gained substantial research interest. A promising solution has emerged as wireless charging of UAVs, which has the capability to replenish the battery over the ether. Wireless charging has several advantages in terms of on-demand availability, usage flexibility, product durability and user convenience [96]. To solve the problem of distance and power for IoT devices and UAVs, and support the paradigm of wireless charging, the distributed laser charging (DLC) system [97] has appeared as an emerging wireless charging technique. 

Distributed laser charging, also known as spatially distributed laser cavity resonance, is a practical light-based charging technique which has recently been commercialized. According to the relevant patent cooperation treaty (PCT) patents (US 9,905,988 B2 and US 9,225,140 B2) [98], we can summarize this technology as follows: retroreflectors in the receiver part-reflect the incident beam from the transmitter and also deflect the transmitted light beam path back to the transmitter. It also allows tunable beam parameters. The power flow due to resonance is stopped when line-of-sight (LOS) between transceivers is blocked. Once this LOS path is restored then optical power transmission is possible at longer distances. 

Besides laser charging, the DLC method guarantees self-alignment to charge UAVs and IoT devices without any special tracking and positioning, as long as transceivers are in line of sight with each other. In addition, WPT of DLC can be stopped after blocking LOS by any intermediate object. A small DLC receiver can be easily mounted in a smartphone or sensor while the transmitter can be installed on a room ceiling like a light bulb. Moreover, a single DLC transmitter is capable of charging multiple devices simultaneously. DLC provides secure charging for UAV and mobile devices with a similar connection such as Wi-Fi. As DLC uses PV cells rather than a collecting lens, it is a practical and cost-efficient solution for UAVs. As equipping UAVs with heavy batteries puts a burden on the UAV, so DLC techniques can be a lightweight solution long to extending the flight time of UAVs. In [99] the authors investigated relationships between energy, power and battery dynamics for a DLC-based quadrotor UAV. 

**Figure 12:**
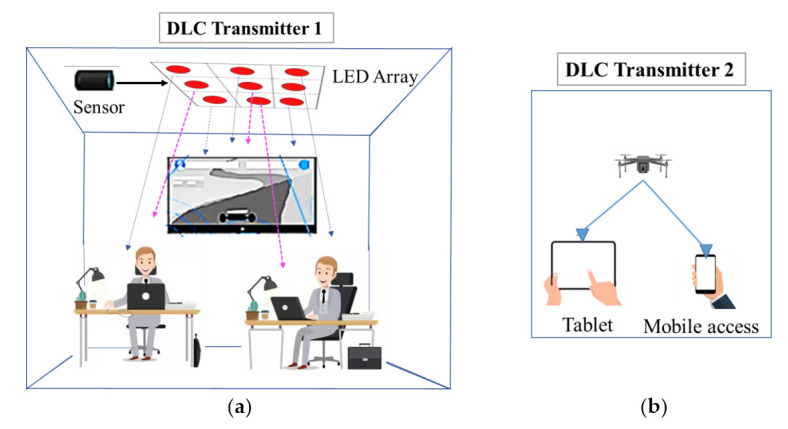
DLC potential applications. (**a**) LED array transmission; (**b**) drone transmission.

Figure 12 presents DLC mechanism, applications and features, etc. It outlines a brilliant idea to charge mobile electronic devices anywhere and anytime. In Figure 12a,b, a DLC transmitter 1 is attached with an LED array to a DLC-equipped light bulb. This DLC transmitter can be easily installed on the ceiling to charge IoT and mobile devices located wihin its range. In the second scenario, a DLC transmitter is attached to a drone which is used to charge IoT devices such as tablets and mobile phones. Meanwhile, the drone is also equipped with a DLC receiver. Thus, it can be charged by DLC transmitter 2. It is worth noting that the efficiency of a DLC system is highly influenced by several factors, e.g., electricity-to-laser and laser-to-electricity conversion efficiency, laser transmission attenuation and laser wavelength. Q Zhang et al. [24] proposed a multimodule DLC model and briefly explained the evaluation of power conversion or transmission for each individual module, considering the impacts of temperature, PV cell, attenuation and laser wavelength. Q. Liu et al. [97] have discussed the DLC method for safe mobile applications. The authors have briefly demonstrated the concept of the DLC technique in the context of smartphone charging. They have proposed two wireless charging techniques: (i) DLC-aided, infrastructure-based network, (ii) DLC-based ad-hoc network. They have demonstrated the potential of the DLC technique through these network architectures to enable a fully charged utopia for smartphones. It can provide safe, mobile, long-distance wireless charging for IoT devices. It also has the significant feature to supporting multiple IoT devices simultaneously. 

DLC has also been used for quadrotor UAVs. It can charge UAVs with its self-alignment feature, thus it does not require specific tracking or positioning as the LoS link is established. DLC receivers can be easily embedded into UAVs due to their small size. These features make DLC suitable for aerial networks. In [100], the authors proposed a simple DLC-source quadrotor UAV model to investigate energy and battery dynamics. In [101], the authors proposed a novel framework for the trajectories of multi-UAVs through the prediction of users’ mobility information. Authors used the DLC method for power control. A compact DLC is embedded in a battery-powered off-the-shelf UAV. As DLC has self-aligning capability and LoS path is also available due to the high altitude of UAVs, thus DLC can charge UAVs as long as they remain in the DLC’s coverage area. DLC-aided UAVs can fly for a long time without landing before any specific maintenance is required. In [102], the authors proposed artificial intelligence for DLC-aided UAVs. In this method, the trajectory of UAVs and model predictive control (MPC), optimal numbers and locations of charging stations can be optimized by using AI solutions. In [103], the authors proposed a novel design of distributed and multi-layer UAVs (DAMU) 5G networks and stated that DLC methods, along with machine learning-assisted computer vision and sensor techniques, can be helpful in ensuring safe laser energy harvesting. Table 4 summarizes a comparison of DLC with different WPT techniques.

### DLC Characteristics

**Figure 13:**
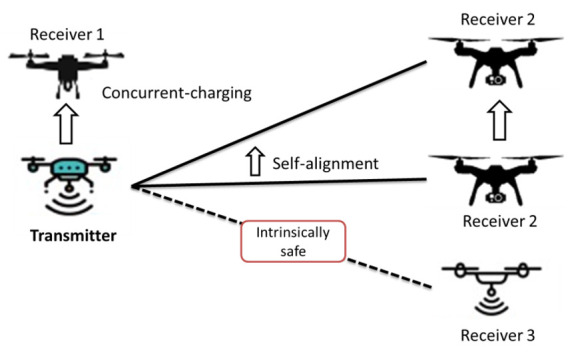
DLC features.

DLC is intrinsically safe, visibility agnostic, self-aligning and can support electromagnetic interference- (EMI) free operations and concurrent multiple-receiver hot-spot charging. DLC offers several benefits over other LPT techniques, such as large transmission distance, compact size and access control. However, DLC also has some critical concerns, including inherent LOS dependence, QoS, laser hazard, power conversion efficiency, propagation attenuation, DLC receiver tracking and DLC transmission distance under path-loss aspects. Some characteristics of DLC are as follows:

(1) *EMI-Free:* There is no leakage of power outside the resonating beam in DLC. In contrast to other WPT techniques, DLC does not impose RF radiations. Thus, it does not inflict EMI on nearby electronic devices.
(2) *Compact Size:* In DLC, the beam diameter is almost one millimeter and the receiver can be compact in size e.g., a smartphone camera.
(3) *Visibility Agnostic:* DLC can depend on visible, as well as ultraviolet (UV) and infrared (IR), lasers. Both IR and UV laser beams are invisible, which is suitable for some applications because it does not impose visible light interference. This visibility agnostic feature of DLC makes it reliable and flexible in different applications.
(4) *Concurrent Wireless Charging:* A DLC system generates a resonating beam when a receiver is exposed to the LOS path of the transmitter. Hence, a single transmitter has the capability to create several resonating beams pointed towards multiple receivers, which enables concurrent WPT from one transmitter to multiple receivers.
(5) *Intrinsically Safe:* The DLC power level can reach up to tens of watts which raises safety concerns. However, the spatially distributed resonator structure of DLC makes it different from integrated resonating lasers. In DLC, when an obstacle comes into the resonating beam path, the laser is curtailed immediately without any additional decision-making circuit.
(6) *Self-Aligning:* In DLC, the charging process continues as the LOS path remains between the transmitter and receiver components. In such a scenario, the distributed resonator is capable of generating a resonating beam without any tracking or aiming feature. No user assistance in starting the charging process in DLC leads to a Wi-Fi-type experience. Several features of DLC. including concurrent charging, self-aligning and intrinsic safety, are shown in Figure 13.

## 6. Simultaneous Wireless Information and Power Transfer (SWIPT)

**Figure 14:**
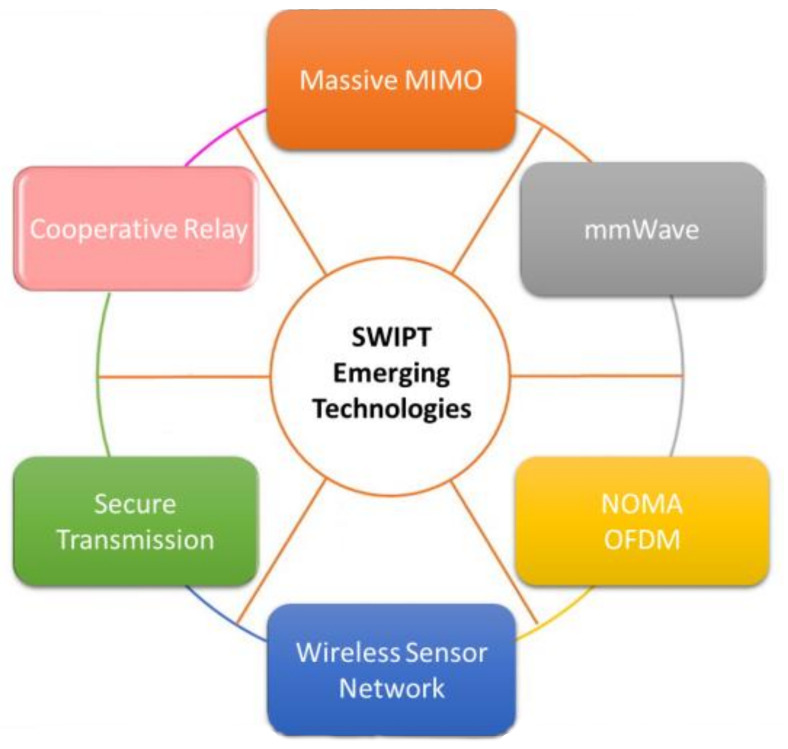
Emerging SWIPT technologies.

Gathering energy from the environment and converting this energy to electrical power has become an interesting research area. This technique is useful in delivering required power to wireless networks from renewable and clean energy resources. Generally, there are two types of energy harvesting methods: environmental and radio frequency. RF-based energy harvesting techniques include a promising method known as wireless power transfer. This technique has been further developed to transmit both energy and information which is known as “simultaneous wireless information and power transfer” (SWIPT). SWIPT plays a crucially important role in devices with limited energy capacity. SWIPT has become attractive through supporting sustainable and ubiquitous energy resources as well as information delivery services. SWIPT-enabled emerging technologies are presented in Figure 14. Early works reported on SWIPT assume that the same signal is used to transfer both energy and information without incurring any losses. To enable SWIPT, we need to split the received signal into two components: one for energy harvesting and other one for information decoding. This splitting can be achieved in different domains such as power, time and antenna [104].

(1) Time switching (TS)

When time switching is used, the receiver switches according to time domain. In this method, the transmitted signals are split in terms of their time domain. As the signal arrives at the first time slot, the signal transmission or decoding information is performed. Simple hardware is used for time switching. However, the TS technique also requires accurate time synchronization and energy/information scheduling [105].

(2) Power splitting (PS)

When power splitting is performed, the receiver is divided into two distinct power levels. The first component is passed to the rectenna circuit for energy harvesting, while second component is used for information decoding. The PS technique is different from TS as it uses a complex receiver and requires the power-splitting factor to be optimized. However, it uses only one time slot to perform energy harvesting and information decoding [106].

(3) Antenna switching (AS)

In the antenna switching technique, antenna elements are used for rectifying and decoding purposes to realize SWIPT. In this technique, the receiver is divided into two components, one is used for energy harvesting while the other is used for information decoding. However, AS techniques require possible solutions to be optimized and suitable for all communications. This problem can be mitigated through MIMO decoding and forward (DF) relaying and dynamic algorithms [107].

**Figure 15:**
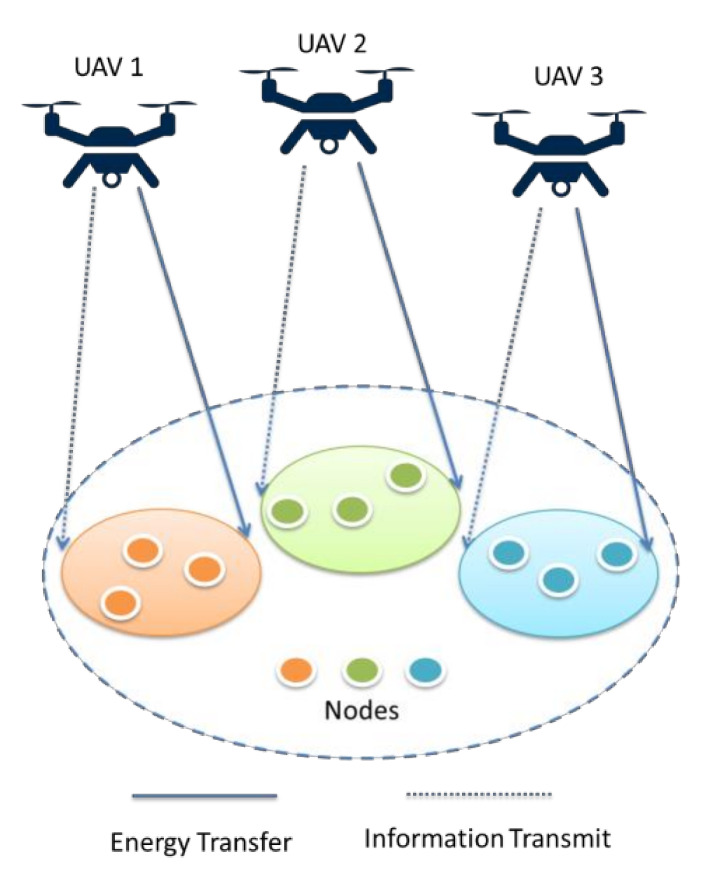
Multi-UAV-enabled SWIPT scenario.

The use of UAVs as aerial wireless communication platforms has gained significant attention due to their high flexibility and adjustable altitude. Therefore, UAV-aided SWIPT can support IoT and future wireless networks. However, there will be several issues involved in implementing multi-UAV-aided SWIPT, as shown in Figure 15. For instance, considering the spatial flexibility of UAVs, the location problem shifts from 2D to 3D deployment [108]. Furthermore, user resource allocation, rate-energy trade-offs and power splitting for each UAV cannot be ignored [109]. Although several studies in the literature have reported on UAV-aided SWIPT, only a few studies are dedicated to UAV-aided SWIPT for IoT. In [110], the authors propose a novel time division multiple access mechanism to reduce energy consumption of the UAV which performs computation, communication and WPT. In [111], the authors established UAV-aided SWIPT for the IoT network for emergency communication in three defined areas: emergency area, wide area and dense area. In the proposed system, a phased antenna array is embedded in a UAV to charge IoT devices. Moreover, an energy harvesting optimization strategy was studied for coverage, charging time and flight altitude of UAV.

Some recent works have focused on mmWave UAV-based relaying systems for SWIPT because it offers benefits such as significant array gains and short-distance transmission. However, it also raises security challenges and physical layer security analysis for mmWave UAV-based relay networks for SWIPT has become an interesting research topic [112]. In [112], the authors proposed a secure transmission technique for mmWave UAV-based relay networks for SWIPT, in which they derived a close-form expression for the lower side of the average capacity rate. In [113], the authors used the power-splitting technique for UAV-aided SWIPT for IoT. The authors designed a mathematical model through an optimization problem which includes the AUV’s speed and power budget constraints. Similarly, the authors in [114] used the time switching technique for a UAV-aided SLIPT system in which source nodes charged the UAV and then the UAV sent the information to the intended receiving node. From these studies, we find that theoretically, TS is a special form of PS based on binary splitting power ratios.

## 7. Simultaneous Lightwave Information and Power Transfer (SLIPT)

Although SWIPT offers the advantage of transmitting information and power simultaneously, it can also be a source of RF pollution and interference of data transmission. One alternative method to avoid RF drawbacks is to harvest energy from lasers and LEDs. Using an optical source, it is possible to simultaneously deliver data and perform energy transfer. Exploiting an optical source to deliver power and carry information is mooted as a key technology to wirelessly recharge the batteries of UAVs, Internet-of-Things (IoT), Intelligent Internet-of-Things (IIoT) and Internet of Underwater Things (IoUT). Researchers extended this concept. naming it simultaneous light wave information and power transfer (SLIPT) [115]. Recently, SLIPT has received significant attention as an effective technique for WPT between communication terminals. It can be a cost-effective solution for wireless systems, e.g., autonomous machines such as UAVs and remote sensors. It is also very promising for aerospace, smart house, healthcare and underwater applications.

**Figure 16:**
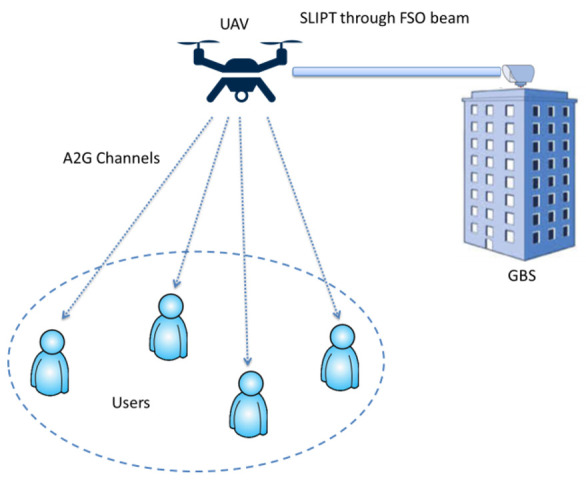
An illustration of A-RAN with SLIPT.

The idea of SLIPT has been demonstrated in indoor IoT applications, powering of IoUT devices and UAVs [116,117]. Recently, J. Fakidis et al. [118] experimentally demonstrated SLIPT for 1 Gbps GaAs VCSEL and a photovoltaic link which is envisaged to be the paradigm for next generation backhaul connectivity. If this strategy is properly optimized, it can provide significant gains in spectral efficiency, power transfer, interference management, energy consumption and time delay. This strategy also generates a remarkable trade-off between rate and harvested energy. SLIPT is also referred to as a promising technological paradigm to unlock UAV-enabled fifth-generation (5G) and beyond backhaul communications. In [117], the authors proposed airborne radio access networks (A-RANs), which is a promising technology due to its capabilities of cost-efficient, fast and on-demand improvement of the current telecommunication infrastructure. The major issues in such networks are reliable links with ground users and energy sustainability of the aerial platforms (APs), such as UAVs, as shown in Figure 16. In this study, the authors proposed a novel approach of using SLIPS and a hybrid FSO/RF relaying protocol. The ground base station (GBS) transmits both power and information to the UAV based on the SLIPT idea, while the UAV connects with the ground users through air to ground (A2G) orthogonal RF channels. In another study [119], the authors studied a UAV-assisted multicasting system using SLIPT technology. In this proposed system, the UAV used the power splitting method to harvest wireless power and decode backhaul information over the FSO link simultaneously. In [120], the authors proposed a novel mechanism to realize energy harvesting using pulse amplitude modulation (PAM). Because SLIPT solutions revolve around time splitting (TS) and average power (AP), the authors compared this mechanism with SLIPT-TS and SLIPT-TS-AP. The presented work shows that optimization can be achieved in a simple way, which can meet the EH design requirements of the UAVs.

In Table 5 different charging techniques for UAVs are shown.

## 8. Potential Challenges in UAV Charging Techniques

Although UAV charging techniques can enhance flight time, and mission duration, there several challenges which could hamper their deployment in the future. In this section, we have shed some light on these challenges.

### 8.1. Efficiency Improvement Issue

The efficiency of laser power transfer is a challenging issue. With the growing developments in LPT, its applications are increasing. The overall efficiency is not sufficient for commercial developments. The research community is working on efficiency improvement for commercial applications. Mainly researchers are focusing on enhancing PV cell efficiency. Meanwhile, researchers have also shown limited interest in optimizing lasers for power beaming.

### 8.2. Safety Concerns

Nowadays, laser technology has emerged from hospitals, and it is available in private enterprises and offices. It is crucially important to establish a laser safe environment. Improper use of laser devices is potentially dangerous. It can cause irreversible injury to the eyes and skin. The biological damage caused by lasers is due to photochemical, acoustical and thermal processes. There is a need to develop stringent safety mechanisms such as scanning LIDAR. Moreover, most efficient lasers operate near IR wavelengths which are in the retinal hazard region. Thus, precautionary measures should be taken to eliminate retinal hazards. Longer wavelengths beyond this region can be an alternative solution. However, this has high cost and low efficiencies. Another major hazard is complacency which it is imperative to consider when designing laser safety perspectives. It involves effective hazard analysis, proper control measures and knowledge of laser standards. It is still unclear that laser-aided UAVs in crowded areas are safe. It can pose environmental challenges and risks for other flying species.

### 8.3. Laser Beam Control Measures

Direct beam exposure can be minimized through proper safety constraints of the laser-aided UAV system. The appearance of any object into the field of view of the laser is critical. Significant time is needed before any object comes into the proximity of the beam path. In such scenarios, proper engineering controls, which can terminate or attenuate the laser beam, are required. Beam control is also essential as the receiver is prone to reflecting some of the incident laser energy. The receiver component includes a gimbal sensing system which can mitigate the reflected energy by redirecting it into an area of safe dissipation. End-to-end and mobile LPT applications should be developed to avoid reflection and redirect this energy into an absorber target to reduce any hazard.

### 8.4. Optical Communication Problem

In future, LPT systems might be able to combine efficient optical communication. By using a PV cell as a receiver for communication, control information and forward command might be delivered, along with power transmission, which could result in a dual-use system. The technique to modulate communication links onto transmitted photon energy will revolutionize robust free space optical communication. However, it is still an open challenge to incorporate communication signals into the optical energy path.

### 8.5. Propagation Losses in the Optical Beam

The performance of optical systems is limited due to three characteristics of free space:

- Water and dust particles which absorb and scatter photons.
- The wave fronts are distorted due to the refraction index of air, which is affected by the gradients in the air caused by temperature and wind.
- Waves expanding beyond the optic’s ability to collimate.

Scattering and absorption coefficients vary according to the airborne particle size. In general, atmospheric articles such as fog, rain and smoke affect beam transmission. In normal conditions, scattering and absorption do not cause severe impacts like distance and atmospheric gradients. Distance is also an important factor which highly influences the beam transmission. Laser mode structure is the largest determinant of beam energy over distance. In such cases, two types of lasers are chosen: single mode and multimode laser. Single mode lasers, such as disk lasers, can be used for LPT up to 1 km. However, multimode lasers cannot even reach the diffraction limit.

### 8.6. The Tracking and Aiming

Tracking and aiming research is a major challenge because some UAVs are flexible and small in size, which creates difficulties in tracking. Moreover, laser beam transmission in the atmosphere causes linear and nonlinear effects. These effects decrease the beam quality and aiming accuracy. Aiming error will cause misalignment; consequently, it will decrease the efficiency of the laser beam and PV cell conversion efficiency. Thus, there is a need to develop high precision tracking and aiming for UAVs.

### 8.7. The PV Panel

At present, commercially available PV cells have low conversion efficiencies of around 20%. If the beam quality is not good, it will significantly reduce the conversion efficiency of PV cells and can damage them There is a need to design a reliable and efficient structure for PV panels which takes beam quality into consideration.

### 8.8. The Maximum Power Point Tracking Algorithm

A nonlinear relationship exists between the voltage and output power of PV panels. There is a specific maximum power point for these devices. Because of irregular illumination, there are several extremes in output characteristic curve of a PV panel. Thus, it is suggested to consider the maximum power point tracking algorithm to enhance the efficiency of PV panels. At present, conventional maximum power point techniques, such as open-circuit voltage, incremental conductivity and perturbation observation are used only for uniform illumination. Therefore, the research fraternity is focusing on maximum power point methods for irregular illumination.

### 8.9. Energy Efficiency

The laser-powered drone technology comes at the expense of several factors. For instance, a significant amount of optical energy is wasted in free space. Due to this feature, laser-powered drones require high power laser sources. It is still unclear that performance gain can justify the additional energy costs. Several industries are investigating methods to enhance the efficiency of LPT by significantly reducing energy losses. Hence, future studies must address this critical challenge and target further improving the PV receiver’s efficiency in harvesting energy from the laser transmitter.

### 8.10. Technological Cost and Complexity

Another concrete challenge in large-scale deployment of laser-powered UAVs is the complexity and cost of manufacturing the entire power transfer system. The technical uncertainties and technological costs of such systems give rise to several concerns about availability and scalability. In our view, this technology is not fully mature yet; thus, further research contributions are required before the large-scale deployment of laser-powered UAVs for civilian applications.

### 8.11. Vulnerability to Weather Conditions

Harsh weather conditions impose critical challenges for laser-powered UAVs. Weather conditions such as cloud, fog, dust, snow, fog, smog, winds and atmospheric turbulence affect the laser beam divergence, alignment and power at the UAV [128]. Thus, future research studies must investigate weather effects and mitigative solutions for UAV laser charging. As wind perturbation can cause misalignment with ground stations, fluctuation and positioning error in UAVs, future research should focus on design mechanisms which can withstand such harsh weather conditions. As laser-powered UAVs are prone to these atmospheric conditions, here rechargeable and tethered UAVs are regarded as energy efficient solutions. Moreover, weather conditions also impact the tracking system, so strategies for efficient real-time tracking of UAV mobility should be investigated.

### 8.12. Lack of Dynamic Power Load Balancing 

There is an overhead in the performance of charging systems, which consequently leads to several challenges in terms of performance degradation, battery degradation and power exhaustion. As a result, the need for efficient charging systems has become a critical challenge [129].

### 8.13. Survivability and Battery Life

UAV lifetime and battery capacity are limited. Therefore, fitting the power must be enhanced to enable starvation and survivability situations. It is a crucial factor which can substantially enhance the UAV lifetime.

### 8.14. Joint Scheduling under Mixed Recharging Mode 

Future UAVs might be powered through different types of energy sources, including battery, fuel cell and solar cell to prolong battery lifetime during persistent tasks. It is essential to effectively manage the UAV’s recharging behaviors under hybrid power supplies. In addition, UAVs can offload some computing tasks to nearby unmanned ground vehicles (UGVs) in order to reduce energy consumption [130]. Thus, the collaborative scheduling of communication, computing and hybrid energy resources between UGVs and UAVs, along with considering their competition and cooperation, becomes a critical challenge.

### 8.15. Fast and On-Demand UAV Recharging

In [130], the authors proposed vehicle-assisted wireless rechargeable UAV networks (VWUNs) which provide a viable approach to support automatic and on-demand recharging services by deploying UGVs with WPT techniques. However, due to limited vehicle roofs, the charging pads installed on UGVs can face capacity limitations. Moreover, as multiple UGVs and UAVs with specific energy requirements can be located in an operational area, their cooperation must be taken into account. Similarly, current works mostly focus on one-to-one charging, while charging multiple UAVs is neglected. Thus, fast recharging methods for multi-UAVs must be designed.

### 8.16. Route and Charging Scheduling of UAVs and UGVs

UAVs with low battery state-of-charge should plan their trajectories on the basis of location, position and altitude in order to reduce battery consumption along with avoiding mission failure and collisions. Thus, efficient and stable charging mechanisms are required to support fast charging. Moreover, considering the high mobility of UGVs and UAVs and the associated network topology, a distributed approach to energy and route scheduling is required to carry out real-time decisions.

### 8.17. Route Planning

By leveraging computer vision, edge computing and machine learning algorithms, collision-free and short trajectories can be adopted to substantially reduce energy consumption. Collaborative routing mechanisms can be used for UAV recharging scheduling. In [131], the authors discussed UAVs for data collection with wireless charging. In the proposed system, UAVs can hover over a static wireless charger which support wireless charging of UAVs whenever UAVs lack in energy. Such systems can support efficient charging, data collection and enhanced throughput. Similarly, by discretizing the UAV trajectories into spatio-temporal segments, efficient charge scheduling methods can be adopted to reduce energy consumption.

## 9. Future Research Directions

Due to their diverse applications in military, remote sensing, traffic monitoring, disaster management, search and rescue, precision agriculture and parcel delivery, UAVs are currently receiving significant attention from both academic researchers and industrial experts. Because of these applications, and to meet extended mission requirements, UAV flight time, battery capacity and payload carrying capability must be improved. Among these, charging the UAV’s battery is critical. Several wired and wireless charging technologies are currently being used to recharge UAVs. Still, the research community is looking for cost-effective, secure and quick ways to recharge UAVs. Contact-based charging, battery swapping, fuel cells and super capacitors are all employed. However, most of these techniques have operational difficulties, high requirements, physical assistance, serious concerns about human injury and electrical hazards during operation. Contactless charging mechanisms, such as wireless power transfer, LPT, DLC, SWIPT and SLIPT, have emerged as promising solutions in this scenario, ensuring the safety of both the human operator and the device equipment. AI, machine learning-based computer vision, smart sensors, Internet of Things (IoT) and integration with novel technologies such as intelligent reflecting surfaces (IRSs), will make UAV technology more efficient, reliable, secure and intelligent. UAV performance can be improved by using smart control mechanisms, self-learning algorithms, tracking and aiming techniques and energy and trajectory optimization. Future research should focus on cooperative algorithms based on multiple UAVs, secure transmission, power allocation design, physical layer (PHY) security in SWIPT-based UAVs, coverage gain via directional antennas and IRS-aided WPT for UAVs, among other topics. Future research will focus on mitigating solutions for attacks such as monitoring attacks, software attacks, interference attacks, safety attacks, spoofing attacks and jamming attacks [6]. In the future, researchers should focus on multi-UAV swarms, UAV-aided secure relaying with cooperative jamming, blockchain and mobile edge computing aided UAVs, cooperative communication with power splitting and MS-aided resource allocation for UAV swarms. In terms of charging, research should focus on pointing and tracking, misalignment, efficient battery management, dynamic charging, fuel cell-based UAVs, supercapacitor-based rapid charging, drone-to-drone charging, efficiency enhancement of PV cells, human safety concerns for laser-powered UAVs and novel charging coil designs.

## 10. Conclusions

The integration of contact-based and contactless charging techniques into UAVs has been a long process. In the context of contactless-based techniques however, there is a research gap with limitless development opportunities. The need for and feasibility of safe and fast charging techniques, such as MRC, CPT, IPT and LPT, will increase as demand for drone services grows. The future of UAVs is dependent on charging techniques that can charge multiple UAVs while saving money, time and stress on operating bodies. This study emphasizes previous research contributions to highlight the need for WPT in UAVs. In addition, a brief history of UAVs, as well as several characteristics and standardizations of UAVs, are discussed. Furthermore, this research covers the technical aspects of WPT techniques for drones by elaborating on existing WPT techniques and how these techniques can significantly improve autonomous operations. This paper comprehensively reviews the advancements in LPT techniques for UAVs, including DLC, SWIPT, SLIPT and related open issues. Finally, the article discusses potential challenges and future research directions. Future work on this research must focus on mathematical modeling of the electromagnetic field in order to investigate the most effective solutions for UAV charging. The research community should concentrate on multi-UAV charging and consider dynamic charging approaches. Optimization methods for UAV coil design, as well as designing multiple coils for a UAV for high power output and energy harvesting, are key research areas.

## Figures and Tables

**Table 1:** Charging techniques to charge UAVs [4].

EMF Based Charging	Charging Type	Non-EMF Based Charging	Charging Type
Capacitive charging	Static charging up to a few mm	Gust soaring	In-flight charging
Inductive charging	Static charging up to a few cm	PV integrated	In-flight charging
Magnetic resonance charging	Static charging up to a few cm	Laser beaming	In-flight charging
		Battery dumping	In-flight charging

**Table 2:** Comparison of different batteries [34].

Characteristics	Li-S	LiPo	Ni-Mh	Ni-Cd
Specific power (W/kg)	600	2800	900	300
Energy density (Wh/L)	350	300	300	100
Specific energy (Wh/kg)	350	180	80	40

**Table 3:** Comparison between batteries and super capacitor [69].

Type	Energy Density (Wh/kg)	Power Density (W/kg)	Cycle Life (Times)	Efficiency of Charging and Discharging (%)	Advantages	Drawbacks
Lead-acid battery	30–40	200–300	300–400	75	High recycle rate, low cost	Poor performance at low temperature
Ni-Mh battery	60–80	800–1500	>1000	75	Long lifespan, high energy density	High manufacturing cost, high self-discharging rate
Li-ion battery	100–120	600–2000	>1000	90	Long cycle life, lightweight, high energy density, high voltage	Security risk, non-overcharge, life reduce at high temperature
Super capacitor	4–15	1000–10,000	>10,000	85–98	Fast charging and discharging speed, pollution-free and extremely long life	Low energy density

**Table 4:** A comparison of different WPT techniques [97].

WPT Technique	Advantage	Disadvantage	Charging Distance	Application
Microwave radiation	Longer charging range	Low charging efficiency, health and safety issues in high exposure	Up to several kilometers	LEDs, implanted body devices, sensors, RFID cards
Magnetic resonance coupling	Non-line-of-sight (NLOS) charging, high charging efficiency, charging multiple devices	Complex implementation, limited charging distance,	Up to a few meters	Electrical vehicle charging, home appliances, mobile electronics
Inductive coupling	Simple implementation, safe	Alignment issues, heating effect, short charging range	Up to a few centimeters	Contactless smartcards, RFID tags, mobile electronics
Distributed laser charging (DLC)	Suitable for mobile applications, SWIPT and LBS ready, visibility agnostic, EMI free, safe, self-alignment	Low charging efficiency, LOS required	Up to several meters	LEDs, sensors, consumer electronics, mobile devices

**Table 5:** A comparison of different UAV charging techniques in various applications.

Reference	Name	Type	Energy Efficiency	Human Intervention	Advantages	Drawbacks
[121]	UGV-assisted WPT	Wireless	Medium	No	On-demand self-recharging No human intervention	Complex route/resource/landing scheduling
[122]	UAV-assisted WPT	Wireless	Medium	No	On-demand self-recharging No need to land	Hard to operate autonomously Prone to aerial collision
[123]	Stationary WPT	Wireless	Medium	No	High charging feasibility No human intervention	Need additional flight
[124]	RE-based charging	Harvesting energy from environment	Medium	No	No need to land No additional flight	Weather-dependent Limited harvested energy Need additional weight and size
[125]	Laser PB charging	Wireless	Low	Medium	No need to land No additional flight	High deployment cost Need complete UAV motion information
[126]	Battery hot swapping	Swap	Very high	Medium	Support multi-UAV charging	High round-trip energy cost Issues in autonomous swapping
[127]	CS-based charging	Wired/wireless	High	Medium	Support multi-UAV charging	High round-trip energy cost Low charging feasibility

## Data Availability

Not applicable.
